# Large-Scale *de novo* Oligonucleotide Synthesis for Whole-Genome Synthesis and Data Storage: Challenges and Opportunities

**DOI:** 10.3389/fbioe.2021.689797

**Published:** 2021-06-22

**Authors:** Li-Fu Song, Zheng-Hua Deng, Zi-Yi Gong, Lu-Lu Li, Bing-Zhi Li

**Affiliations:** ^1^Frontiers Science Center for Synthetic Biology and Key Laboratory of Systems Bioengineering (Ministry of Education), Tianjin University, Tianjin, China; ^2^School of Chemical Engineering and Technology, Tianjin University, Tianjin, China; ^3^LC-BIO Technologies Co., Ltd., Hangzhou, China

**Keywords:** oligonucleotide synthesis, DNA synthesis, synthetic biology, whole-genome synthesis, data storage in DNA, DNA-based data storage

## Abstract

Over the past decades, remarkable progress on phosphoramidite chemistry-based large-scale *de novo* oligonucleotide synthesis has been achieved, enabling numerous novel and exciting applications. Among them, *de novo* genome synthesis and DNA data storage are striking. However, to make these two applications more practical, the synthesis length, speed, cost, and throughput require vast improvements, which is a challenge to be met by the phosphoramidite chemistry. Harnessing the power of enzymes, the recently emerged enzymatic methods provide a competitive route to overcome this challenge. In this review, we first summarize the status of large-scale oligonucleotide synthesis technologies including the basic methodology and large-scale synthesis approaches, with special focus on the emerging enzymatic methods. Afterward, we discuss the opportunities and challenges of large-scale oligonucleotide synthesis on *de novo* genome synthesis and DNA data storage respectively.

## Introduction

*De novo* oligonucleotide synthesis (oligo synthesis) is the synthesis of defined sequences of single-strand nucleic acids. Its early efforts could be found out in the 1950s, followed by large-scale automation in the 1980s (Kosuri and Church, 2014). Starting in the early 1990s, the microchip-based large-scale oligo synthesis methods were developed (Fodor et al., 1991; Pease et al., 1994; Singh-Gasson et al., 1999; Gao et al., 2001; Ghindilis et al., 2007; Ma et al., 2012; Kosuri and Church, 2014). To clarify, “large-scale oligo synthesis” here refers to the parallel synthesis of massive oligos with various sequence contents. The massive and cheap oligos produced by large-scale oligo synthesis have a wide range of applications. For examples, the oligos can be used as molecular tools for the construction of large cell populations with various genotypes (Kosuri and Church, 2014). It also can be used for understanding and engineering of regulatory elements, proteins, genetic networks and metabolic pathways (Kosuri and Church, 2014). Due to the wide application potentials, large-scale oligo synthesis have greatly helped with the life science studies in many aspects, e.g., synthetic biology, protein engineering, genome engineering, metabolic engineering and so on (Ma et al., 2012; Kosuri and Church, 2014; Casini et al., 2015). Among these applications, *de novo* whole-genome synthesis and DNA data storage are drawing more and more attention (Cello et al., 2002; Church et al., 2012; Gibson, 2014; Kosuri and Church, 2014; Shen et al., 2017; Wu et al., 2017; Xie et al., 2017; Zhang et al., 2017; Kohman et al., 2018; Lee et al., 2018). Despite the remarkable progress that large-scale oligo synthesis has achieved in the past decades, substantial further improvements are still highly demanded for emerging wide applications. However, due to the natural limitations of chemical reactions, it is a challenge to further improve the phosphoramidite chemistry-based methods in terms of accuracy and length dramatically. The recently emerged enzymatic methods with advantages of mild conditions, fast coupling, no hazardous waste generation, show potentials in the realization of next-generation oligo synthesis technologies (Hoff et al., 2019; Lee et al., 2019). In this mini-review, we discuss the current status of large-scale oligo synthesis technologies with special focus on the recent innovations of the enzymatic methods. Then we discuss its applications in *de novo* whole-genome synthesis and DNA-based data storage. The different development routes to meet the future requirements of the two distinct applications will be discussed respectively.

## Current Status of Phosphoramidite Chemistry-Based Oligo Synthesis

**FIGURE 1:**
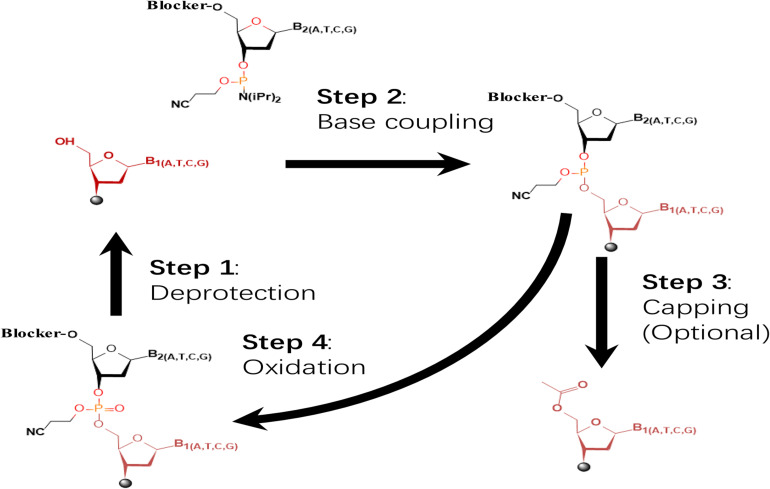
The dominating four-step, phosphoramidite chemistry widely applied in commercial oligo synthesizers. The initial nucleoside(s) is tethered to specific substrate via its 3′ hydroxyl. The synthetic cycle involves four steps of deprotection, base coupling, capping, and oxidation. Synthesis proceeds in direction of 3′ to 5′.

In the past few decades, the solid-phase phosphoramidite method has been the primary choice for most commercial oligo synthesizers (Roy and Caruthers, 2013; Kosuri and Church, 2014). This method was developed by Beaucage and Caruthers in 1980s (Beaucage and Caruthers, 1981; Caruthers, 1985). The four-step synthesis cycle of this method is illustrated in Figure 1. First, the dimethoxytrityl group (DMT) of the nucleotide immobilized on the solid phase is detached by acid catalysis. Second, the 3′ hydroxyl group of the nucleotide to be added in is activated by phosphoramidite and mixed with the tetrazole activator, and the obtained nucleoside-phosphite activator is 5′ hydroxyl-activated nucleotides undergo condensation. Third, a small number of 5′ hydroxyl-activated nucleotides not involved in the condensation reaction are prevented from participating in the reaction by acetylation. Fourth, oxidation of trivalent phosphotriester to pentavalent phosphotriester using an iodine solution. After addition of all nucleosides in series from 3′ to 5′, the obtained oligo is released. Currently, the length of oligos synthesized by the phosphoramidite chemistry-based methods is limited within 200 nucleotides in general and cannot exceed 300 nucleotides theoretically (Palluk et al., 2018). For construction of long DNAs, DNA assembly is required to assemble the short oligos into long DNAs (Casini et al., 2015).

### Column-Based Oligo Synthesis

Column-based oligo synthesis is the first-generation technology based on phosphoramidite chemistry. In this technology, oligo synthesis is carried out separately in the columns. The reagents are pumped through the columns, enabling the iterative addition of nucleotides in a programmable way. Over the past decades, advances in materials, automation, procedure, and purification have led to the synthesis of 100 nt at cost of $0.05 to $0.15 per nt, with an error rate of 1/600 or less (Kosuri and Church, 2014). The commercially available column-based oligo synthesizer can synthesize 96–768 oligonucleotides each containing 10 nmol to 2 μmol at the same time. Column-synthesized oligonucleotides have been used as modules for DNA constructions by assembly methods in previous studies (Ma et al., 2012). However, column-based oligonucleotide synthesis cannot satisfy the requirements of large-scale DNA synthesis in the era of synthetic biology, due to the limitations of low throughput and high cost (Ma et al., 2012).

### Microchip-Based Oligo Synthesis

Starting in the 1990s, oligo synthesis gradually developed into a higher throughput manner by parallel synthesis on a silica surface (Fodor et al., 1991; Pease et al., 1994). This microchip-based (also called ‘microarray-based’) large-scale oligo synthesis technologies, provides an inexpensive source of oligo building blocks for various applications (Ma et al., 2012; Kosuri and Church, 2014). Surpassing the column-based synthesis in terms of throughput and cost, microchip-based oligo synthesis has received wide attention. The commercially available microchip-based synthesizers are based on principle of phosphoramidite chemistry with slight modifications. The major differences between various microchip-based methods are the different mechanisms that applied in steps of deprotection and base coupling (Ma et al., 2012). At present, there are light control, electrochemical, and inkjet printing methods.

The light control methods use light to control the deprotection process, including photolabile 5′ protecting groups (PPG, NimbleGen/Affymetrix) and photo-generated acid deprotection (LC Science). The basic strategy for PPG-based synthesis is to illuminate the surface of a PPG modified solid support with a mask to generate free hydroxyl groups for the coupling of the 3′-*O*-phosphoramidite activated deoxynucleoside. After coupling and capping, the surface of the solid support is illuminated through a new mask, exposing the next active hydroxyl group and coupling with the second 3′-*O*-phosphoramid-activated deoxynucleoside (Fodor et al., 1991; Pease et al., 1994). The expensive masks applied and long synthesis time limits its applications. Later, Singh-Gasson et al. (1999) reported a mask less array synthesis method in which the expensive masks were replaced with Digital Micromirror Device (DMD). The DMD forms an ultraviolet image on the surface of a glass support, enabling selective deprotection. This DMD-based method avoids the need for photolithographic masks, reducing the cost and time (Singh-Gasson et al., 1999). Differently, Gao et al. (2001) used photo-generated acids (PGAs) to activate deprotection reaction of the 5′-OH group to achieve microchip-based synthesis. The 5′-OH group is formed in the subsequent chain extension reaction and coupled with the introduced monomer (Gao et al., 2001). The electrochemical methods (CombiMatrix/CustomArray) use the adjacent anode and cathode electrodes to produce a deprotected active material, which is coupled to a silicon plate for the synthesis reaction in order to selectively deprotect the DMT protection group to control the synthesis of the desired oligonucleotide in specific positions (Egeland and Southern, 2005; Ghindilis et al., 2007). Compared to the PPG and PGA-based methods, more precise and complexed semiconductor fabrication is essential. In addition to the methods as described, several other extensions and changes in microfluidics have also been reported, but have not been widely available or commercialized (Ma et al., 2012). The inkjet printing method was developed by Agilent. In this method, a commercial inkjet printer head was utilized to deliver the phosphoramidite monomer to a specific location on a silicon surface, enabling programmable and parallel synthesis of massive oligos.

Although microchip-based oligo synthesis is more prone to errors due to the heterogeneity and edge effects of the microchip, it has enabled high-fidelity synthesis of oligo pools ∼300-mer after procedure optimization (Ma et al., 2012; Kosuri and Church, 2014). In general, the costs of microchip-based oligo synthesis are 2–4 orders of magnitude cheaper than the column-based oligo synthesis. The cost per nucleotide is between $0.00001 and 0.001 (Kosuri and Church, 2014).

## The Emerging Enzymatic Oligo Synthesis Methods

Enzymatic *de novo* synthesis of oligonucleotide can be dated back to 1955 (Grunberg-Manago et al., 1955). However, due to technical limitations, the earlier studies on enzymatic methods are quite limited (Grunberg-Manago et al., 1955; Severo Ochoa, 1961; Bollum, 1962; Mackey and Gilham, 1971). Since 2016, several studies proved the feasibility of enzymatic oligo synthesis using template-independent or dependent polymerases (Mathews et al., 2016, 2017; Palluk et al., 2018; Hoff et al., 2019). Harnessing the power of enzymes, there are several advantages of enzymatic oligo synthesis methods that compared with chemical synthesis: (1) The enzymatic synthesis reaction is carried out under hydrated and mild conditions, coupled with the specificity of the enzyme, the enzymatic synthesis can reduce the formation of by-products and the depurination of DNA and other damages, so that longer oligonucleotides can be synthesized directly; (2) Synthesis can start from natural DNA (i.e., DNA without protecting groups); and (3) Protein engineering (such as directed evolution or rational protein design) can be used to optimize the system, which cannot be achieved using organic chemistry alone (Mathews et al., 2016, 2017; Palluk et al., 2018; Hoff et al., 2019). Here, we review the recent emerged enzymatic oligo synthesis methods that utilizes either template-independent or dependent polymerases.

### Template Independent Polymerase-Based Methods

**FIGURE 2:**
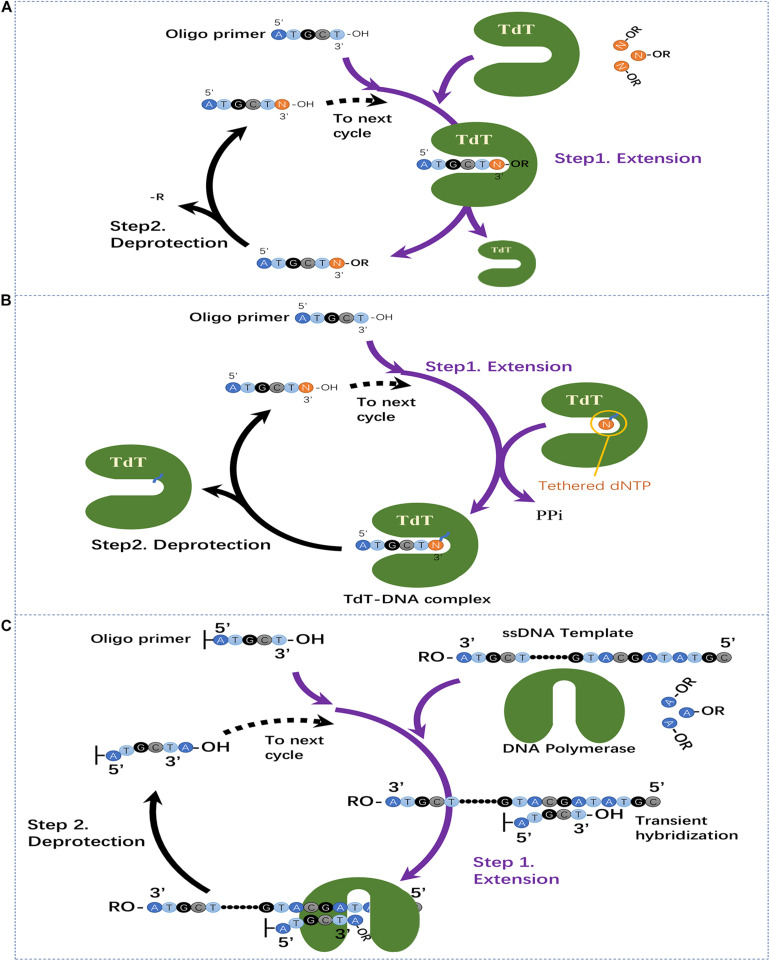
Three recently emerged enzymatic oligo synthesis methods. **(A)** Two-step extension synthesis of DNA oligo using TdT and dNTPs with reversible blocking groups. **(B)** Two-step extension synthesis of DNA oligo using TdT–dNTP conjugates for reversible termination of oligo elongation. **(C)** Template-dependent polymerase mediated oligo synthesis by transient hybridization and chemically blocked substrates.

Utilization of template-independent polymerase for oligo synthesis can be dated back to 1992 (Hyman, 1992). Ramadan et al. (2004) verified that several DNA polymerases, and TdT showed template-independent DNA polymerase activities (Ramadan et al., 2004). The crystal structure of TdT was solved by Delarue et al. (2002). The predominant activity of TdT is addition of deoxynucleotide triphosphates to the 3′ end of DNA. This makes TdT an ideal candidate for usage in oligo synthesis (Motea and Berdis, 2010; Palluk et al., 2018). Although the natural features of TdT are favorable for the implementation of the enzymatic oligo synthesis method, no concrete method using TdT was established until 2017 (Palluk et al., 2018). Early efforts focused on a strategy of using 3′-*O*-modified nucleotides with blocking groups, which have been successfully applied in DNA sequencing by synthesis strategy (Ruparel et al., 2005; Wu et al., 2007; Bentley et al., 2008; Mathews et al., 2016, 2017). By developing the methods for synthesis and purification of 3′-*O*-caged 2′-deoxyribonucleoside triphosphates, Mathews et al. (2016, 2017) demonstrated the feasibility of light-mediated deprotection for enzymatic oligo synthesis using TdT. As shown in Figure 2A, the proposed synthesis cycle using TdT and dNTPs with 3′-blocked groups contains two steps. Step 1 – Extension: the solid-phase immobilized oligo primers are mixed with TdT and specified dNTP with 3′-*O*-blocking groups, then the TdT will catalyze the coupling of dNTP with the oligo primers. Due to the existence of the 3′-*O*-blocking groups, the elongation will stop after coupling of one base. Step 2 – Deprotection: the 3′-*O*-blocking groups of the solid-phase immobilized oligo primers are removed for extension of next base. The coupling time was indicated to be around 60 min and a 4 mer oligonucleotide was synthesized using this method (stepwise yields not reported) (Mathews et al., 2016, 2017). This method suffers from the obstacle that 3′-*O*-modified nucleotides cannot be efficiently incorporated by TdT since there is almost no room for 3′-OH modifications in the nucleotide position of TdT structure (Palluk et al., 2018). Although it has been proposed that protein engineering with TdT can potentially improve its incorporation efficiency (Efcavitch and Sylvester, 2015), no further efforts have been reported.

To overcome the limitations of TdT with 3′-blocked dNTPs, Palluk et al. (2018) established a novel strategy by using TdT–dNTP conjugates for suspending of oligo elongation in each synthesis cycle. In this strategy, a TdT molecule and a dNTP molecule are conjugated to each other, and the dNTP molecule can be added to the primer by the conjugated TdT. When the bound dNTP is added, the 3′ end of the primer is still covalently bound to TdT, blocking the random addition of other TdT–dNTP molecules. This blocking effect is then released by breaking the bond between the TdT and the nucleotide allowing subsequent extension (Palluk et al., 2018). As shown in Figure 2B, the synthesis process contains two major steps. Step 1 – Extension: the oligo primer is exposed to excess TdT-dNTP conjugates and the tethered nucleotide is incorporated into the 3′ end. The covalently attached conjugate can block the extension of other TdT-dNTP molecules. Step 2 – Deprotection: The cleavage reagent cleaves the bond between the incorporated nucleotide and TdT, and the primer is released for subsequent extension. After optimization, the coupling time of this method was around 10–20 s.

### Template Dependent Polymerase-Based Methods

Until very recently, Hoff et al. (2019) reported a novel enzymatic oligo synthesis method using template-dependent polymerase including DNA polymerases and reverse transcriptase. They achieved the single-base extension using oligo that can instantaneously hybridizes to adjacent strands using as few as two binding bases. When multiple transient hybridizations templating different bases are possible, DNA polymerase and reverse transcriptase can then extend the DNA strand. The oligo sequence to be synthesized then can be controlled by adding the desired base. As shown in Figure 2C, specific single strand DNAs (ssDNA) serve as templates and the surface-bound oligo is extended by sequential polymerase-based incorporation of 3′-reversibly blocked nucleotides. Specifically, there are two steps in each extension cycle for the synthesis of arbitrary oligo sequences: Step 1 – Extension: the ssDNA template, polymerase and desired dNTP with 3′-blocking groups are added and the polymerase will extend one base by transient hybridization of the surface-bound oligonucleotide to ssDNA template. Step 2 – Deprotection: the 3′-blocking groups of surface-bound oligonucleotide is removed for next base extension; A 20-base oligonucleotide was successfully synthesized utilizing this approach with stepwise efficiency more than 98% and coupling time around 1 min.

### Future Developments of Enzymatic Oligo Synthesis

Currently, enzymatic oligo synthesis methods are still in their methodology developing stage. The capacities of currently available enzymatic oligo synthesis methods reported in peer-reviewed articles were summarized in Table 1, which includes coupling time, stepwise yield, maximal length achieved and substrate requirements, *etc.* Despite preliminary results from proof-of-concept studies, the coupling time of enzymatic methods was shown to be much shorter than the chemical methods. As preliminary results of proof-of-concept studies, the stepwise yield and maximal length of enzymatic methods are still below the phosphoramidite method. It needs to be clarified that these are results reported in peer-reviewed papers. Several enzymatic oligo synthesis start-up companies, including DNA Script, Molecular Assemblies, Nuclera, Ansa Biotechnologies, Camena Bioscience and Kern Systems also have reported their progress on enzymatic oligo synthesis in public media (Eisenstein, 2020). DNA Script claimed to achieve a step-wise yield of 99.7% and successful synthesis of a 280-mer oligo. Camena even stated that they can produce 300-mer oligo with a step-wise yield greater than 99.9%. Currently, no microchip-based enzymatic *de novo* oligo synthesis has been reported either in peer-reviewed articles or in public media. Nevertheless, the knowledge obtained during optimization of the chemical methods can be very helpful to speed up the development process of microchip-based enzymatic synthesis.

**TABLE 1:** Current capacities of enzymatic oligo synthesis methods reported in literatures in comparison with the dominating phosphoramidite chemistry methods.

	Coupling time	Step-wise	Maximal length	Substrates	Enzyme	Template
	**(per base)**	**Yield**	**achieved**			**requirements**
Reversible terminator	60 min	Not available	4 mer	Purified 3′-blocked dNTP	TdT	No
TdT-dNTP conjugator	10∼20 s	97.7%	10 mer	Purified TdT-dNTP complex	TdT	No
Transient hybridization	1 min	98.4%	20 mer	Purified 3′-blocked dNTP	Template dependent DNA polymerase	Yes
Phosphoramidite chemistry	∼4∼10 min	99.5%	300 mer	Purified 5′-blocked dNTP	NA	NA

*To clarify, these results are based on peer-reviewed articles. Step-wise yield greater than 99.9% and maximal length around 300 mer have been reported by start-up companies in public media. NA, not applicable.*

## Emerging Applications

The massive and cheap oligos produced by large-scale oligo synthesis can be used for many purposes, enabling numerous interesting applications. For example, the oligos can be used for the constructions of large cell populations or DNA variations, which are beneficial in engineering regulatory elements, genetic networks, metabolic pathways, and DNA origami (Kosuri and Church, 2014). This list is only growing with the future developments and innovations relevant to this technology. A previous review has discussed these applications in detail (Kosuri and Church, 2014). Here, we focus on the two attractive applications of *de novo* synthesis of whole genomes and DNA-based data storage.

### *De novo* Synthesis of Whole Genomes

“What I cannot create, I do not understand,” as implied by the famous quote by Richard Feynman, our ability to create and build arbitrary DNA constructs can greatly boost our capacity in understanding the mechanisms of biological systems. Genomes are the whole blueprints of all living matter on earth. The progress of whole-genome synthesis technology has fundamental impacts on life science in many aspects (Cello et al., 2002; Gibson, 2014; Shen et al., 2017; Wu et al., 2017; Xie et al., 2017; Zhang et al., 2017). We will briefly review the history of whole-genome synthesis, and outline the key technologies and workflow involved. We then discuss the future developments to meet the requirements of whole-genome synthesis.

#### Brief History of *de novo* Whole-Genome Synthesis

*De novo* synthesis of genomes offers the capability of complete control over the genetic code of an organism. Due to the small size of viruses and their important roles in the advancement of health and biotechnology, great progress has been made in viral genome synthesis. In 2002, Eckard Wimmer’s research team was the first to generate infectious poliovirus by synthesizing complete cDNA (Cello et al., 2002). After that, humans have reconstructed dozens of RNA viruses through chemical methods, including the Spanish influenza virus in 1918 (Tumpey et al., 2005) and many others (Kosuri and Church, 2014). Several DNA bacteriophages were *de novo* synthesized as well (Smith et al., 2003; Liu et al., 2012). In addition to the viral genome, the Venter Institute has also designed, constructed, assembled and transplanted a fully synthetic bacterial genome to encode viable organisms. In 2008, the 583 kb genome of *Mycoplasma genitalium*, the smallest prokaryotic genome in nature, was chemically synthesized (Gibson et al., 2008). In 2010, The 1.08 Mb Mycoplasma genome was artificially synthesized (Gibson et al., 2010). The first eukaryotic gene combination project (Sc2.0) started in 2011 and has now completed the synthesis and assembly of chromosomes 2, 3, 5, 6, 10, and 12 (Dymond et al., 2011; Annaluru et al., 2014; Mercy et al., 2017; Mitchell et al., 2017; Richardson et al., 2017; Shen et al., 2017; Wu et al., 2017; Xie et al., 2017; Zhang et al., 2017). Recently, Pelletier et al. (2021) constructed a synthetic minimal cell with only 480 genes, which can achieve normal division and proliferation.

#### General Workflow of Whole-Genome Synthesis

**FIGURE 3:**
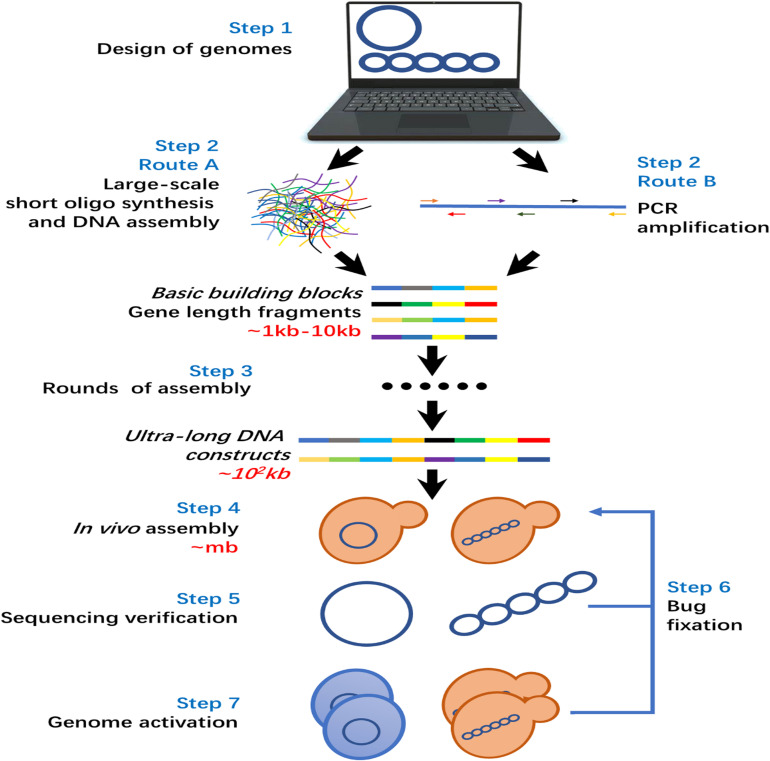
General workflow of *de novo* whole-genome synthesis.

The general workflow of *de novo* genome synthesis is illustrated in Figure 3. Step 1, the genome sequence to be synthesized is designed on a computer with the help of various genome design tools (Lee et al., 2013). Step 2, generation of gene length builds blocks for genome assembly, and there are two optional routes in this step. In route A, the large-scale oligo synthesis is utilized for the generation of huge amounts of short DNA fragments as starting materials for the synthesis of a whole genome. The short oligos are then assembled into gene-length DNA fragments by DNA assembly and ligation. For route B, target gene-length fragments are generated by PCR amplification in using primers produced by traditional column-based oligo synthesizer. In early studies of *de novo* genome synthesis, route B is preferred (Cello et al., 2002; Gibson et al., 2008, 2010). Owe to the fast developments in large-scale oligo synthesis, route A became more advanced in terms of cost and labor intensity and has been applied widely in recent studies (Shen et al., 2017; Wu et al., 2017; Xie et al., 2017). Step 3, the generated gene-length DNA fragments go through several rounds of DNA assembly in a trial-and-error way to generate ultra-long DNA fragments. The assembly rounds required mainly depend on the genome size and applied assembly methods. There is a general trend that the cost and efforts required rises exponentially with the increased size of the genome to be synthesized (Gibson, 2014; Casini et al., 2015; Kohman et al., 2018). Step 4, the ultra-long DNA fragments are assembled into the final genome-size. Due to the low efficiency of *in vitro* assembly of long DNA fragments, *in vivo* assembly is the general choice in most previous studies (Gibson, 2014; Shen et al., 2017; Wu et al., 2017; Xie et al., 2017). Step 5, the assembled genome was sequenced to check for errors/bugs. In case of critical errors/bugs occurring, a bug fixation step (Step 6) was applied to fix them. Step 7, the synthesized genome is activated in a proper host cell. If the activation fails, a bug fixation step is also required to remove the bugs until the synthesized genome was activated successfully. It is worth to emphasize that ‘bug fixation’, i.e., correction of the errors, is crucial for whole genome synthesis. The rapid developing CRISPR/Cas9 base editing tools, which has been widely used for correction of disease associated mutations, have promise in fixing the bugs (Ravindran, 2019).

#### Future Developments of Large-Scale Oligo Synthesis for Whole-Genome Synthesis

Nowadays, all the techniques required to synthesize a whole-genome are already available as shown in Figure 3, especially those with small genome size such as bacteria genomes. However, the cost to synthesize a whole-genome is still very high due to the chemical reagent consumption during oligo synthesis, as well as sequencing validation and error correction steps during oligo assemble steps. Certainly, the large-scale oligo synthesis technology will be improved toward the aspects of low cost, high accuracy, and longer length. This will achieve a cheaper source of oligos as basic step in building blocks for genome synthesis. Meanwhile, the time and labor-intensive DNA assembly and testing process consumes a major part of the costs (Ma et al., 2012; Boles et al., 2017; Kohman et al., 2018) which cannot be benefited through advanced oligo synthesis technology. Thus, in order to reduce the total cost fundamentally, all the functions, e.g., oligo synthesis, DNA assembly, DNA analysis, that are required by whole genome synthesis should be integrated into one platform by using modern automation technologies. A pioneering study toward this direction shows dramatically reduced cost and time required to synthesize a genome (Boles et al., 2017).

### Dense and Long-Term Data Storage in DNA

The early efforts of storing artificial information in DNA can be found since 1996 (Davis, 1996). Limited by the high-cost and low-throughput oligo synthesis and sequencing technologies of the time, only a minimal amount of information was stored (Davis, 1996; Clelland et al., 1999; Bancroft et al., 2001). Church et al. (2012) published a milestone study in using microchip-based oligo synthesis and next-generation sequencing for data ‘writing’ and ‘reading’ of digital data in DNA. This opens up a new window in using synthesized DNA as massive data storage media (Church et al., 2012). Indeed, with outstanding features of long-term, high-density, and low maintain cost, DNA is believed to be a viable and compelling alternative to traditional storage media and to be potential to solve the world crisis of digital data explosion (Church et al., 2012; Zhirnov et al., 2016; Ceze et al., 2019; Ping et al., 2019). The long-term, high-density and low maintenance cost features of data storage in DNA has drawn attention of companies, universities and research institutes world-wide (Goldman et al., 2013; Gabrys et al., 2015*;* Grass et al., 2015; Yazdi et al., 2015; Holmes, 2016; Erlich and Zielinski, 2017; Choi et al., 2018; Jensen et al., 2018; Organick et al., 2018; Song and Zeng, 2018; Anavy et al., 2019; Chen K. et al., 2019; Lee et al., 2019; Tomek et al., 2019).

Data storage in DNA is a technology of “storing information in polymers” which can be dated back to the 1980s when Kaempf et al. (1987) proposed the concept. This technology was termed as “molecular data storage” in later studies and has been an active and challenging area since then (Boukis and Meier, 2018; Martens et al., 2018; Ceze et al., 2019; Meier and Barner-Kowollik, 2019). Unlike the traditional planner media which requires a prepared surface for data writing, and molecular data storage requires precise polymer synthesis and sequencing methods for ‘writing’ and ‘reading’ of information instead. Furthermore, the synthesized polymers can be simply mixed to distribute the encoded information in a three-dimensional (3D) space. This extra dimension allows the molecular data storage to be an ultra-high-density storage technology outpacing the traditional planner media which are only two-dimensional (2D) (Boukis and Meier, 2018; Martens et al., 2018; Song and Zeng, 2018; Ceze et al., 2019; Meier and Barner-Kowollik, 2019). DNA is the natural formed polymers which are utilized as information carrier by all living matter on earth. With tremendous availability in enzymes that can synthesize, replicate and even repair DNA molecules, DNA and its derivatives have significant advantages over other types of polymers for data storage purposes, at least for now. In this section, we will briefly summarize the recent studies on DNA data storage technology followed by discussions on the future developments of large-scale oligo synthesis technology for data storage purposes. For readers who are interested in other types of molecular data storage can refer to the recent review by Meier and Barner-Kowollik (2019).

#### Principle of Data Storage in DNA

**FIGURE 4:**
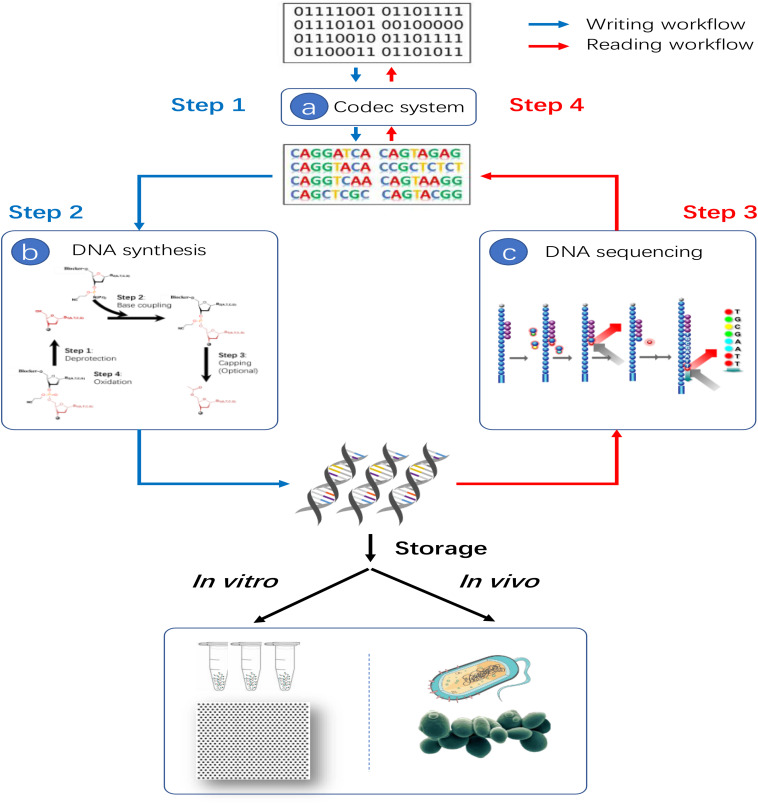
Basic principle of data storage in DNA. Three key technologies are required: **(a)** Codec technology which can encode binary string into DNA string and decode DNA string back to binary string. **(b)** DNA synthesis technology for the actual data writing process. **(c)** DNA sequencing technology for data reading process.

Although reliable data writing and reading with massive error-rich DNA molecules are complex and challenging, the basic principle of data storage in DNA is not complicated. As shown in Figure 4, the four steps are involved in the process of data storage in DNA generally. At first, the digital information, represented by string(s) of “0” and “1,” which is transformed into DNA string(s) of “A,” “T,” “G” and “C” with redundancy codes added by a well-designed codec system. Then, a DNA synthesis device is utilized to synthesis the DNA string(s) into actual DNA molecules, accomplishing the data-writing process. Next, the DNA molecules are sequenced by a DNA sequencer in order to read out the information in forms of DNA string(s). Finally, the DNA string(s) are decoded into the original binary information by the codec system with error correction codes corresponding to the applied redundancy codes in the first step. There are three key technologies required for data storage in DNA: (A) codec system, (B) DNA synthesis technology, (C) DNA sequencing technology as shown in Figure 4. In addition, depending on the storage vessel of actual DNA molecules, additional steps to modify and transfer the synthesized DNA molecules into a proper niche, i.e., tubes, plates or even living cells, may also be required.

#### Recent Progress of Data Storage in DNA

**FIGURE 5:**
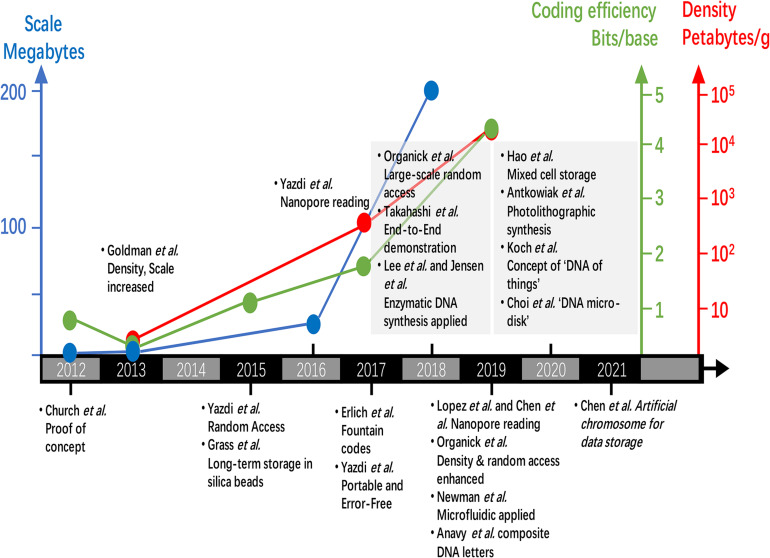
Recent progress of data storage in DNA on data scale, coding efficiency, data density and novel applications.

The key achievements of data storage in DNA since 2012 is presented in Figure 5. Shortly after the proof concept of milestone study by Church et al. (2012), researchers of European Bioinformatics Institute developed a base-3 Huffman code system that significantly increased the scale, density, and reliability of data storage in DNA (Goldman et al., 2013). In 2015, by storing data-encoding DNA strands in silica beads, Grass et al. (2015) achieved reliable data storage in DNA, which was estimated be stable for more than 2000 years. This strategy was later improved in order to increase the storage density (Chen W. D. et al., 2019). Also in 2015, Yazdi et al. (2015) established a DNA-based storage architecture that enables random access of data blocks for the first time. The next year (2016), Tabatabaei Yazdi et al. (2016) applied Nanopore sequencer for reading of DNA encoded data and considering its low-cost and pocket-size advantages, proving the concept of portable DNA data storage. Later in 2017, by applying fountain codes, Erlich and Zielinski (2017) established an architecture which achieves ultra-high coding efficiency (bits per base) and enables PCR-based copy free DNA data storage. Organick et al. (2018) constructed a random access approach which can work on large data sets of more than 200M. However, this approach was proved to work on even larger and higher density data sets later (Organick et al., 2019). Takahashi et al. (2018) developed a complete end-to-end DNA data storage device. Song and Zeng (2018) established an *in vivo* coding system which enables ultra-long-term short message storage in the DNA of living cells. Lopez et al. (2019) established a DNA assembly strategy to overcome the problem in reading short DNA strands by Nanopore sequencer. Chen K. et al. (2019) developed a unique way of using Nanopore sequencer by encoding information in different DNA hairpins instead of nucleic acid bases. Newman et al. (2019) applied digital microfluidic to automatically retrieve data from high-density DNA data storage library. Anavy et al. (2019) implemented a high coding efficiency of 4.29 bits per base by using composite DNA letters. The recent review by Ping et al. (2019) has described these progress in detail which includes a comprehensive summary of the evolving error correction codes. In brief summary, great efforts have been focused on improving the accuracy, scale, coding efficiency and density of data storage in DNA by construction of error correction codes and integration with oligo synthesis and DNA sequencing technologies. The data scale has increased from the 0.66 MB (megabytes) in 2012 to 200 MB in 2018 (Church et al., 2012; Organick et al., 2018). The storage density has increased from 2.2 PB/g (petabytes per gram DNA) to 17,000 PB/g (Erlich and Zielinski, 2017; Organick et al., 2020). The coding efficiency has increased from 1 to 4.29 bits per base (Anavy et al., 2019). Meanwhile, the function of data copy (Erlich and Zielinski, 2017), random access (Yazdi et al., 2015; Organick et al., 2018), long-term stability (Grass et al., 2015; Chen W. D. et al., 2019) have also achieved outstanding outcomes.

Besides the rapid evolving codec systems, the novel storage modes and applications were also proposed. Koch et al. (2020) proposed a DNA-of-things storage architecture to create materials with embedded memory. Choi et al. (2020) proposed DNA micro-disks concept for the efficient management of DNA-based data storage by QR-coded micro-sized disks carrying data-encoded DNA molecules. Furthermore, several recent studies have also proved that the feasibility of massive data storage in DNA of living cells. Chen et al. (2021) encoded the information in a synthetic yeast artificial chromosome with four autonomously replicating sequences and maintained good stability during yeast replication. Yim et al. (2021) used electrical signals to regulate redox reactions in cells, thus causing changes in plasmid copy number, and then used it to control the trapping frequency of different sequences by Cas proteins and to achieve one-step storage of data in cells. Hao et al. (2020) assembled a large oligo pool into vector plasmids based on homology and introduced them into bacterial cells for mixed culture, then combining the advantages of *in vivo* cell systems and *in vitro* data storage mediated by oligo pools to achieve high-fidelity replication of DNA molecules at low cost.

#### Future Development of Large-Scale Oligo Synthesis for DNA-Based Data Storage

The development of cheaper, longer, and higher throughput oligo synthesis technologies would be necessary for DNA-based storage. While previous studies achieved substantial progress on data scale, stability, random access and copy free, *etc.*, the cost, especially the synthesis cost, emerged as one of the key obstacles for practical data storage in DNA (Ceze et al., 2019; Ping et al., 2019). It was estimated that 7–8 orders magnitude decrease of the synthesis cost is essential for DNA data storage to outpace the currently used magnetic tape based on storage technology. And, another obstacle to current data storage technology is bandwidth (throughput of data writing and reading). Without an accordant writing and reading bandwidth, the storage capacity of DNA is not feasible and cannot be utilized to solve the world crisis of digital data explosion. There is a six orders-of-magnitude gap for DNA synthesis to catch up with mainstream cloud archival storage system. In order to support low-cost and high bandwidth data storage in DNA, the large-scale oligo synthesis technology needs to be dramatically improved in terms of cost, speed, and throughput. Driven by the demands of life science, the throughputs of DNA synthesis technology will be persistently improved via more parallel synthesis regardless of DNA data storage application. Also, increased parallelism could be carried out via more supporting microarray or plate area and smaller spot size. The smaller spot size can also save the reagent consumption proportionally as discussed previously (Ceze et al., 2019). Although the throughput and cost gap seem immense, there are many aspects that can be optimized to reduce the cost of oligo synthesis technology for data storage purposes. The-state-of-the-art oligo synthesis technologies are designed for life science, where the accuracy of synthesized DNA molecules is highly desired. Unlike life science, with the incorporation of error-correcting codes, DNA data storage requires much lower accuracy of synthesized DNA strands. Thus, the expensive and labor-intensive validation and error correction steps could be avoided. The synthesize steps can be simplified and the high purity reagents used can be replaced with low purity ones to reduce costs. For example, the capping step is applied to reduce the deletion errors and can be removed which may increase the synthesis speed, length limitation and reduce the cost in principle. High purity reagents are much more expensive than the low purity ones. The state-of-the-art large-scale oligo synthesis platforms use super high purity (>99.99%) dNTP reagents. For data storage purposes, the dNTP reagents with 99% purity or even lower may be acceptable which may greatly reduce the cost. Otherwise, the newly emerged enzymatic DNA synthesis methods using template-independent TdT or using template-dependent DNA polymerase can provide another route for development of high-throughput and low-cost oligo synthesis technology for data storage purposes (Palluk et al., 2018; Hoff et al., 2019). Indeed, two groups of Jensen et al. (2018) and Lee et al. (2018) have already applied TdT-based enzymatic DNA synthesis method to data storage applications. In their study, they applied a similar strategy of using terminator free TdT enzymes for DNA data storage purpose. Random identical bases were added sequentially and the transition of base types was utilized for data recording. It is worth mentioning that, in the study by Lee et al. (2019), the terminator free TdT-based DNA synthesis was proved to be a superior combination with Nanopore sequencer which is optimal for sequencing of long DNA strands with high error rate.

In addition to the fundamental technical developments regarding the cost and throughput, the oligo synthesis technology should be integrated with sequencing technology as well as other essential automatic functions to accomplish the full function of data writing, copying, reading, and random access in one device as the proof-of-concept study by Takahashi et al. (2018). For *in vivo* DNA data storage, additional function models for handling of cell cultivation, DNA assembly and transformation are also required. Finally, four new synthetic nucleic acids: 6-Amino-5-nitropyridin-2-one (Z), 5-Aza-7-deazaguanine (P), Isocytosine (S), and Isoguanine (B) are reported (Hoshika et al., 2019). Incorporation of these new bases would further increase the coding efficiency in the near future (Ping et al., 2019).

## Conclusion

Our chase to more advanced large-scale oligo synthesis technology in terms of cost and throughput is ‘endless’ which is similar to the endless demands of faster processors in the electronic industry. Despite the astonishing achievements on large-scale oligo synthesis that have been made in the past decades, our capacity to write DNA sequences still lags far behind our ability to read them. While the rapid development of DNA sequencing technology has heavily relied on the “sequencing by synthesis” method which can utilize the power of naturally formed polymerase for sequencing purpose, the oligo synthesis is dominated by chemical synthesis method with several limitations like limited synthesis length and environmentally hazardous, *etc.* The emerging enzymatic methods uncovers an exciting route for pushing large-scale oligo synthesis technology into next-generation by harnessing the power of naturally formed polymerases. Given that the numerous advances of mild conditions, no generation of hazardous waste, no requirements of protecting groups, *etc.*, we believe that the further optimization of enzymatic methods and its microchip-based parallelization will potentially drive the large-scale oligo synthesis technology into next-generation which is bound to be more accurate, cheaper, faster, and be able to synthesize longer sequences.

The future developments in large-scale oligo synthesis technology to whole-genome synthesis and data storage are both expected to be focused on the automation integration of required functions. For whole-genome synthesis, a fully automated device which can integrate large-scale oligo synthesis with DNA assembly and DNA analysis, and would dramatically reduce the cost and labor intensive process of genome assembly (Boles et al., 2017). For DNA-based data storage, the large-scale oligo synthesis technology is desired to be integrated with DNA sequencing technology to achieve data “writing” and “reading” in one device. Furthermore, the functions of “random access,” “data copying” also needs to be implemented with modern automation technologies on this integrated platform. In detail, the future adaptions of large-scale oligo synthesis technology for genome synthesis and data storage are believed to have slightly different focus while they both strive for cost controls and more parallel oligo synthesis technique. For oligo synthesis, accuracy and cost are two contradictory factors which means increasing one factor will bring down another one when using the same technique. For *de novo* whole-genome synthesis, accuracy is always the priority focus. This is because the debugging cost is much higher than the benefits that we can get from an inaccurate synthesis process. Thus, the developments of large-scale oligo synthesis for genome synthesis, we must guarantee a high accurate process while improving throughput and reducing cost. At the same time, the DNA assembly and bug fixation technologies should also be developed. Differently, for the DNA-based data storage, the accuracy requirement is much lower compared to the genome synthesis application since the error correction codes could be integrated to correct the errors that emerge. Instead, data storage application requires much higher throughput and lower costs. Thus, there is a trade-off between the accuracy and the cost-throughput. The work of Lee et al. (2019) is a perfect case study of such adjustments between the cost and accuracy. More studies following this strategy in the future are expected to reduce the synthesis cost of data storage in DNA for several orders which should be greatly helpful to make the DNA-based storage a practical reality.

## Author Contributions

L-FS and Z-HD wrote the first draft. B-ZL supervised the whole study. Z-YG, L-LL, and B-ZL edited, revised, and finalized the text. All authors contributed to the article and approved the submitted version.

## Conflict of Interest

L-LL is an employee at LC-BIO Technologies (Hangzhou) Co., Ltd. The remaining authors declare that the research was conducted in the absence of any commercial or financial relationships that could be construed as a potential conflict of interest.
